# Supplementary material for: Highly-conducting molecular circuits based on antiaromaticity
Source: Nat Commun. 2017 Jul 19;8:15984. doi: 10.1038/ncomms15984 (PMC5524926; doi:10.1038/ncomms15984)
Supplement: Supplementary Information [file ncomms15984-s1.pdf]

Type of file: PDF
Size of file: 0 KB
Title of file for HTML: Supplementary Information
Description: Supplementary Figures, Supplementary Notes, Supplementary Tables and Supplementary References

Type of file: PDF
Size of file: 0 KB
Title of file for HTML: Peer Review File
Description:

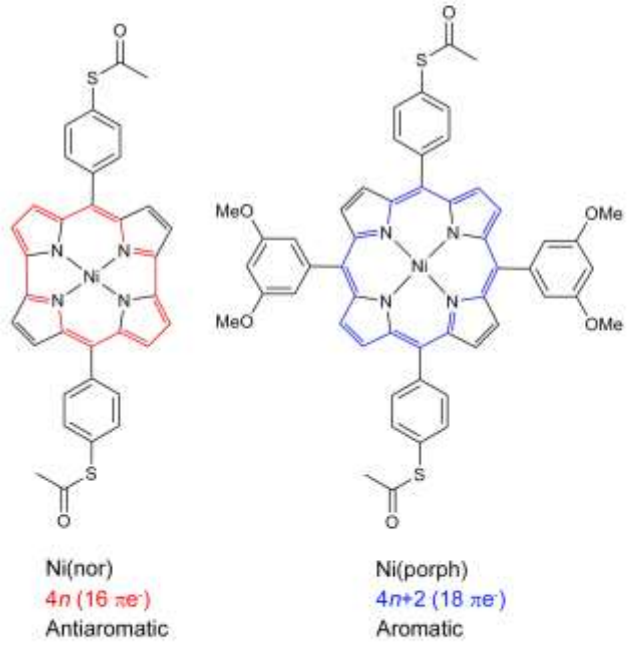

Supplementary Figure 1 | Molecular structures of the antiaromatic Ni(nor) and its aromatic counterpart Ni(porph).

Supplementary Note 1. Synthesis and characterization of Ni(nor) and Ni(porph)

1-1. Synthesis of Ni(nor)

Preparation of 4-(methylthio)benzaldehyde. A toluene solution (150 mL) of 4-(methylthio)benzaldehyde (10 mL, 71.4 mmol) was placed in a round bottom flask, equipped with a dean-stark trap. Neopentyl glycole (11.14 g, 107.1 mmol) and *p*-toluenesulfonic acid (89 mg, 650 μmol) were added to the solution. The solution was refluxed for 2 hours. The resulting solution was cooled down to room temperature, washed with an aqueous $NaHCO_3$ solution, dried over Na_2SO_4, evaporated to remove the solvent, and purified by recrystallization using CH_2Cl_2 and hexane. Finally, 4-(methylthio)benzacetal was obtained as a white powder in 83% yield (14.1 g). ^{1}H NMR (300 MHz, $CDCl_3$: δ = 7.26 ppm) δ = 7.42 (2H, d, J = 8.4, Ph-H), 7.26 (2H, d, J = 8.4, Ph-H), 5.36 (1H, s, acetal-H), 3.76 (2H, d, J = 10.5, acetal-CH_2), 3.64 (2H, d, J = 10.5, acetal-CH_2), 2.47 (3H, s, *S*-CH_3), 1.29 (3H, s, acetal-CH_3), and 0.80 (3H, s, acetal-CH_3).

Preparation of 4-(S-acetylthio)benzaldehyde. A dimethylacetamide (DMA) solution (50 mL) of 4-(methylthio)benzacetal (2.38 g, 10 mmol) and $NaSCH_3$ (3.5 g, 50 mmol) was heated to 140 °C overnight under anaerobic conditions. The resulting solution was cooled down to room temperature and acetyl chloride (3.57 mL, 50 mmol) was added dropwise to the solution. The resulting mixture was poured into water and extracted with diethyl ether. The organic phase was then washed with water, and dried over Na_2SO_4. The yellow solution was concentrated upon solvent removal. To the concentrated solution, hexane was added to produce white crystals of 4-(S-acetylthio)benzaldehyde in 98.9 % yield (2.63 g). ^{1}H NMR (300 MHz, $CDCl_3$: δ = 7.26 ppm) δ = 7.55 (2H, d, J = 8.4, Ph-H), 7.42 (2H, d, J = 8.4, Ph-H), 5.41 (1H, s, acetal-H), 3.78 (2H, d, J = 11.1, acetal-CH_2), 3.65 (2H, d, J = 11.1, acetal-CH_2), 2.40 (3H, s, acetyl-CH_3), 1.28 (3H, s, acetal-CH_3), and 0.80 (3H, s, acetal-CH_3).

Preparation of p-(S-acetylthio)phenyl-2,2'-dipyrromethane. A CH_2Cl_2 solution (1 mL) of *p*-(S-acetylthio)benzacetal (700 mg, 2.63 mmol) was cooled down to –30 °C under anaerobic conditions and 2 mL of trifluoroacetic acid (TFA) was added to the solution. During the addition, the transparent solution turned pale. The solution was stirred overnight at –5 °C and added to a CH_2Cl_2 solution (20 mL) of pyrrole (50 mL) previously cooled down to –5 °C under anaerobic conditions. After stirring for 30 min, the resulting solution was poured in

water and extracted with CH_2Cl_2. The organic phase was thoroughly washed with water (10 mL × 10) and dried over Na_2SO_4. Solvent was evaporated and the residue purified by column chromatography on silica gel using neat CH_2Cl_2. Evaporation of the appropriate fractions yielded dipyrromethane as a yellow sticky solid (243 mg, 0.82 mmol, 31% yield). ^1H NMR (300 MHz, $CDCl_3$: δ = 7.26 ppm) δ = 7.95 (2H, bs, NH), 7.35 (2H, d, *J* = 8.1, Ph-H), 7.25 (2H, d, *J* = 8.1, Ph-H), 6.71 (2H, bs, pyrrole-H), 6.16 (2H, d, *J* = 2.1, pyrrole-H), 5.91 (2H, bs, pyrrole-H), 5.50 (1H, s, sp^3-H), and 2.42 (3H, s, acetyl-CH_3). ^{13}C NMR (75.5 MHz, CDCl3: δ = 77 ppm) δ = 194.6 (acetyl, CO), 143.7 (phenyl, CS), 134.4 (phenyl, CH), 131.8 (pyrrole, C), 129.2 (phenyl, CH), 126.0 (phenyl, C), 117.4 (pyrrole, CH), 108.2 (pyrrole, CH), 107.3 (pyrrole, CH), 43.5 (sp^3CH), and 30.1 (acetyl, CH_3).

Preparation of 1,9-dibromo-5-[p-(S-acetylthiophenyl)]dipyrrin. *p*-(*S*-acetylthio)phenyl-2,2'-dipyrromethane (157.9 mg, 0.53 mmol) was dissolved in THF (20 mL) under N_2. The solution was cooled down to –75 °C and N-bromosuccinimide (NBS) (190 mg, 1.06 mmol) was added to the solution in two times (100 mg /90 mg, respectively) in 30 min term. The solution was stirred for 1 h before 2,3-dichloro-5,6-dicyano-*p*-bezoquinone (DDQ) (121 mg, 0.53 mmol) was added. The solution was warmed up to room temperature and the solvent evaporated. The residue was purified by means of column chromatography using silica gel and CH_2Cl_2. The first red fraction containing the final product was collected and further purified by recrystallization from CH_2Cl_2/hexane. The final product was obtained as a reddish powder in 67.5% yield (161 mg, 0.36 mmol). ^1H NMR (300 MHz, $CDCl_3$: δ = 7.26 ppm) δ = 12.25 (1H, bs, NH), 7.48 (4H, m, Ph-H), 6.47 (2H, d, *J* = 4.2, pyrrole-H), 6.34 (2H, d, *J* = 4.2, pyrrole-H), and 2.48 (3H, s, acetyl-CH_3).^{13}C NMR (75.5 MHz, CDCl3: δ = 77 ppm) δ = 193.0 (acetyl, CO), 140.0 (pyrrole, CBr), 137.9 (phenyl, CS), 136.4 (sp^2C), 133.5 (phenyl, CH), 131.3 (phenyl, CH), 130.0 (pyrrole, CH), 129.9 (pyrrole, C), 129.6 (phenyl, C), 120.6 (pyrrole, CH), and 30.28 (acetyl, CH_3).

Preparation of Ni(II) complex of 1,9-dibromo-5-[p-(S-acetylthiophenyl)]dipyrrin. A MeOH solution (10 mL) of $Ni(OAc)_2 \cdot 4H_2O$ (89 mg, 0.36 mmol) was added to a CH_2Cl_2 solution (20 mL) of 1,9-dibromo-5-[*p*-(*S*-acetylthiophenyl)]dipyrrin (161 mg, 0.36 mmol) under N_2. The resulting solution was stirred for 1h. Upon solvent evaporation, greenish black crystals were formed. Addition of MeOH (20 mL) was used to complete the crystallization process. The crystals were collected by filtration and dried under vacuum (79% yield, 135 mg, 0.14 mmol).

Preparation of Ni(Nor). $NiCl_2 \cdot 6H_2O$ (253 mg, 0.178 mmol), triphenyl phosphine (538.4 mg, 1.935 mmol), and Zn powder (68 mg, 0.98 mmol) were dissolved in dimethylformamide (DMF) (72 mL) under N_2. The solution was heated to 50 °C for 1.5 h and cooled down slightly. When the temperature reached 45 °C, a DMF solution of Ni(II) 1,9-dibromo-5-[*p*-(*S*-acetylthiophenyl)]dipyrrin (170 mg, 0.178 mmol) was added and the resulting solution stirred for 30 min at that temperature. The solution was cooled down to 0 °C and filtered to remove the Zn powder. The filtrate was washed with cold water many times to remove the DMF solution, extracted with ethyl acetate, dried over $NaSO_4$, and evaporated to remove the majority of the solvent. The residue was then sonicated in hexane. After filtration, the filtrate containing triphenyl phosphine was removed. The solids were further purified by column chromatography on silica gel using a CH_2Cl_2/hexane mixture as eluent. Recrystallization from CH_2Cl_2/hexane yielded Ni(Nor) (9% yield, *ca.* 10 mg, *ca.* 0.0156 mmol). ^1H NMR (300 MHz, $CDCl_3$: $\delta = 7.26$ ppm, 1.29×10^{-3} M) $\delta = 6.73$ (4H, d, $J = 8.4$, Ph-H), 6.06 (4H, d, $J = 8.4$, Ph-H), 2.29 (4H, d, $J = 4.2$, βH), 2.23 (6H, s, acetyl-CH_3), and 1.91 (4H, d, $J = 4.2$, βH).

1-2. Synthesis of Ni(porph)

Preparation of 3,5-dimethoxyphenyl-2,2'-dipyrromethane. A solution of 3,5-dimethoxybenzaldehyde (500 mg, 3.0 mmol), pyrrole (30 mL), and CH_2Cl_2 (20 mL) cooled down to –10 °C under N_2. To the solution, trifluoroacetic acid (TFA) (2 mL) was added and stirred together for 20 min. The reaction was quenched by addition of an aqueous NaOH solution. The final mixture was extracted with CH_2Cl_2, and the organic phase dried over anhydrous $NaSO_4$. The solvent including unreacted pyrrole was then removed by evaporation followed by vacuum distillation. After silica gel column chromatography using CH_2Cl_2 and recrystallization in CH_2Cl_2/hexane, the target compounds was obtained as a white powder in 49% yield (412 mg, 1.46 mmol). ^1H NMR (300 MHz, $CDCl_3$: $\delta = 7.26$ ppm) $\delta = 7.94$ (2H, bs, NH), 6.69 (2H, dd, $^{d}J = 4.2$, $^{dd}J = 2.7$, βH), 6.39 (2H, d, $J = 2.4$, Ph-H), 6.36 (1H, t, $J = 2.1$, Ph-H), 6.15 (2H, dd, $^{d}J = 3.0$, $^{dd}J = 6.0$, βH), 5.96 (2H, bs, αH), 5.41 (1H, s, sp^3H), and 3.74 (6H, s, methoxy-CH_3).

Preparation of the free-base porphyrin in Ni(Porph). A CH_2Cl_2 (0.5 mL) solution of *p*-(*S*-acetylthio)benzacetal (260 mg, 0.977 mmol) was cooled down to –5 °C under N_2.

Trifluoroacetic acid (TFA) (0.5 mL) was then added and the resulting solution was stirred overnight at –5 °C. Separately, a CH_2Cl_2 solution (100 mL) of 3,5-dimethoxyphenyl- 2,2'-dipyrromethane (276 mg, 0.977 mmol) was cooled down to –10 °C under N_2. The first solution was added to the second and stirred together for 1.5 h. Finally, 2,3-dichloro-5,6-dicyano-1,4-benzoquinone (DDQ) (333 mg, 1.47 mmol) was added and the solution stirred overnight. Column chromatography on silica gel using CH_2Cl_2 gave three different porphyrin derivatives formed through scrambling condensations. The target free-base porphyrin was isolated from the second column fraction and further purified by recrystallization in CH_2Cl_2/hexane (19%, 62.6 mg). ^{1}H NMR (300 MHz, $CDCl_3$: δ = 7.26 ppm) δ = 8.96 (4H, m, βH), 8.85 (4H, m, βH), 8.28 (4H, d, J = 8.1, Ph-H), 7.82 (4H, d, J = 8.1, Ph-H), 7.40 (4H, d, J = 2.1, Ph-H), 6.91 (2H, t, J = 2.1, Ph-H), 3.97 (12H, s, methoxy-CH_3), 2.62 (6H, s, acetyl-CH_3), and –2.82 (2H, bs, NH). ^{13}C NMR (75.5 MHz, CDCl3: δ = 77 ppm) δ = 193.9 (acetyl, CO), 158.9 (phenyl, CO), 143.9, 143.8, 143.2, 135.2 (phenyl, CH), 132.5 (phenyl, CH), 130.9, 128.9, 127.8, 120.1, 119.8, 118.9, 113.9 (phenyl, CH), 100.2 (phenyl, CH), 55.6 (methoxy, CH_3), and 30.5 (acetyl, CH_3).

Preparation of Ni(porph). A toluene solution (10 mL) of the free-base porphyrin (15.5 mg, 0.0176 mmol) and $Ni(acac)_2$ (5 mg, 0.0195 mmol) was refluxed overnight under N_2. The solution was cooled down and directly embedded in a silica gel column. The target complex Ni(Porph) was obtained using CH_2Cl_2 as the eluent. Further purification was done by recrystallization in CH_2Cl_2/hexane (78%, 10 mg). ^{1}H NMR (300 MHz, $CDCl_3$: δ = 7.26 ppm) δ = 8.86 (4H, dd, ^{d}J = 5.1, ^{dd}J = 2.7, βH), 8.74 (4H, dd, ^{d}J = 5.1, ^{dd}J = 2.7, βH), 8.06 (4H, d, J = 8.1, Ph-H), 7.73 (4H, d, J = 8.1, Ph-H), 7.20 (4H, d, J = 2.4, Ph-H), 6.83 (2H, t, J = 2.1, Ph-H), 3.92 (12H, s, methoxy-CH_3), and 2.58 (6H, s, acetyl-CH_3). ^{13}C NMR (75.5 MHz, CDCl3: δ = 77 ppm) δ = 193.9 (acetyl, CO), 159.1(phenyl, CO), 142.5, 142.4, 142.0, 134.4 (phenyl, CH), 132.7 (phenyl, CH), 132.5, 132.3, 132.1, 127.8, 119.0, 117.9, 113.0 (phenyl, CH), 100.1 (phenyl, CH), 55.6 (methoxy, CH_3), and 30.4 (acetyl, CH_3).

^{1}H NMR spectrum of Ni(Nor) showed distinctantiaromatic features. Pyrrole protons are resonated as two doublets at 2.29 and 1.91 ppm. Theantiaromatic nature of the norcorrole core in Ni(Nor) was confirmed by Nucleus Independent Chemical Shift calculations (see Supplementary Fig. 2). Two reversible oxidation potentials are observed at 0.838 V and 0.302 V for Ni(Nor) and 0.798 V and 0.578 V for and 0.838 V for Ni(Porph), respectively. Only one reduction curve of Ni(Porph) was detected at –1.71 V, while two reversible

reduction curves are observed at –0.718 V and –1.406 V, respectively. These reduction potentials are rather higher than dimesitylnorcorrole Ni(II), of which the reduction potentials were measured at –0.913 V and –1.674 V, resulting in a smaller HOMO–LUMO gap. The electrochemical HOMO–LUMO gaps are 1.083 V, 2.288 V, and 1.020 V for Ni(Nor), Ni(Porph) and dimesitylnorcorrole Ni(II), respectively.

Supplementary Note 2. Ni(nor) Nucleus Independent Chemical Shift (NICS) calculations

Calculations were carried out using the Gaussian 09 program. Full optimizations were performed without any symmetry restriction with Becke's three-parameter hybrid exchange functional and the Lee–Yang–Parr correlation functional (B3LYP) and a basis set consisting of Lanl2dz for Ni and 6-31G(d) for the rest. The NICS values were obtained with the GIAO method at B3LYP/631Lanl2dz level on the optimized geometry.

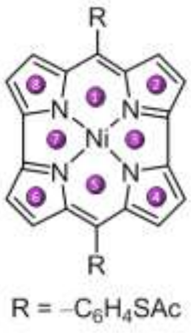

Ghost atom	*NICS(0)*$_{iso}$
1	42
2	5
3	34
4	5
5	42
6	5
7	40
8	4

Supplementary Figure 2 | NICS values calculated for Ni(nor). Ghost atoms probe the contribution of each individual ring in the norcorrole macrocycle. Positive values denote paratropic ring currents characteristic of antiaromatic systems.

Supplementary Note 3. STM-BJ conductance studies

A series of single-molecule studies have recently reported a negative correlation between aromatic resonance energy and molecular conductance.[1-4] In other words, non-aromatic compounds are more conductive than their aromatic counterparts. Although the origin of this counterintuitive fact is a matter of debate, these results suggest that antiaromatic compounds may display exceptional charge transport capabilities. In that respect, the effect of the antiaromaticity on the charge transport properties was explored using molecules with aromatic, nonaromatic and partially antiaromatic characters, where the effect was found inconclusive due to the presence of counterbalancing electronic effects arising from the distortion of the molecular backbone and the electronic interaction between the molecule and the metal electrodes. [3] [5] Hence, the charge transport capabilities of antiaromatic compounds remain to be explored.

Compounds for the present conductance studies (Scheme 1) were designed to enable direct comparison, with comparable dimensions $d_{S\text{-}S}$~1.7 nm and bearing thioacetate (−SAc) molecular linkers to assist in the junction formation. Thioacetates offer a number of advantages with respect to more the conventional terminal thiols (−SH). Their chemical robustness facilitates their synthesis, handling and storage in ambient conditions. The acetyl groups bind to Au electrodes through the S lone pair, a strategy which has been shown to result in selective bonding and clear experimental conductance signatures.[6] The acetyl groups were reported to spontaneously cleave on the Au surface forming Au-S bonds when a gold substrate was exposed to a high concentrated solution (*ca.* 40 mM) of molecules bearing the acetyl groups for long enough periods of time.[7] [8] Our results with dilute molecular deposition conditions (0.1 mM) suggest that in this case the whole thioacetate groups are preserved.

3-1. STM-BJ conductance-distance traces

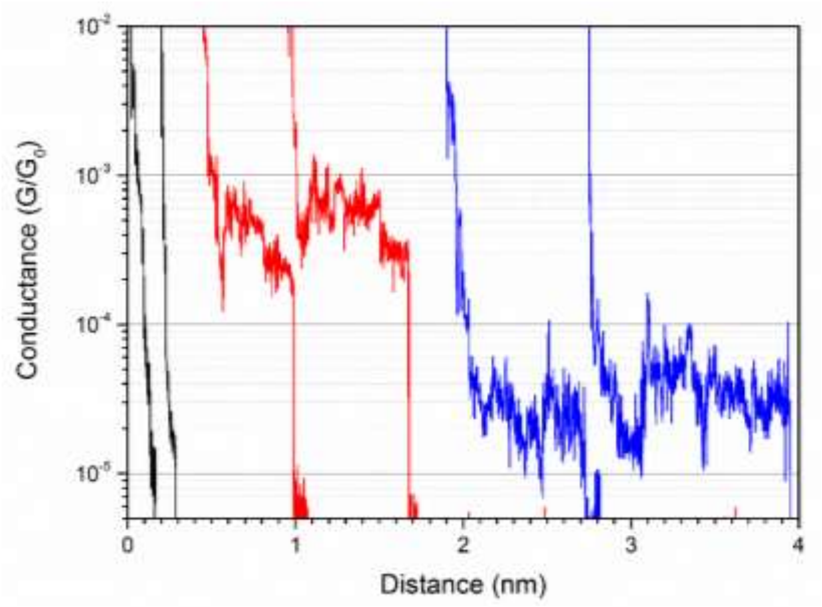

Supplementary Figure 3 | Representative examples of STM-BJ traces, showing a conventional tunnelling decay (black), Ni(nor) molecular plateaus in the 10^{-4} G_0 region (red), and molecular plateaus in the 10^{-5} G_0 region characteristic of the aromatic complex Ni(porph) (G_0 = 77481 nS).

3-2. Additional 1D STM-BJ histograms

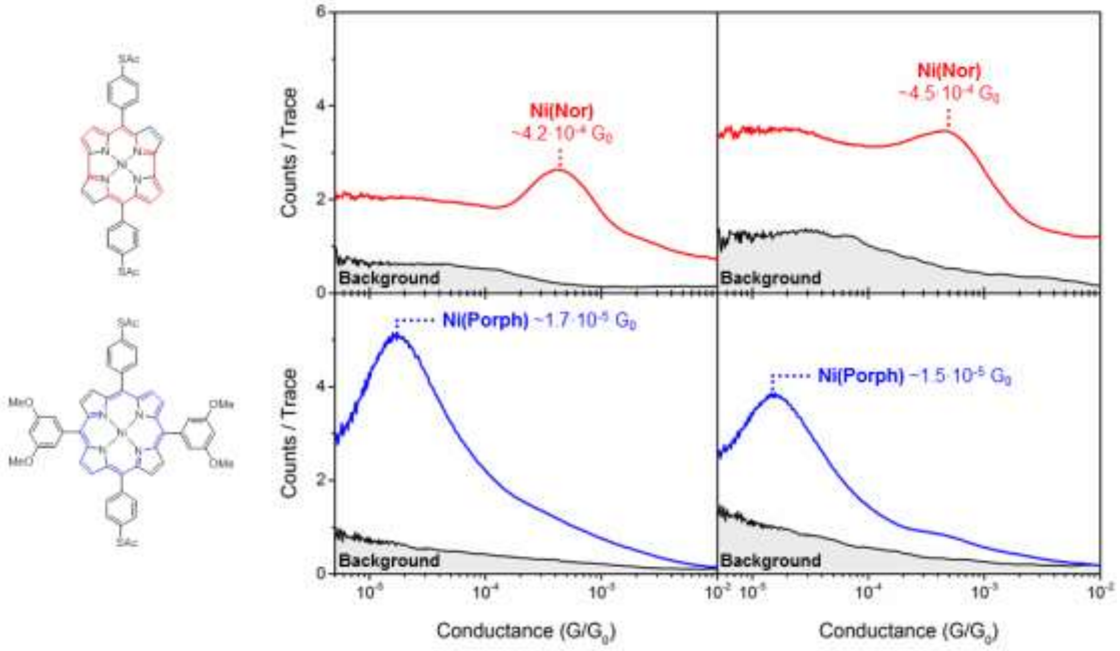

Supplementary Figure 4 | Logarithmically binned conductance histograms of Ni(nor) and Ni(porph), obtained from two analogue samples at 100 mV in air. The so-called background histograms were obtained from the freshly annealed Au(111) substrates prior to molecular deposition. Each individual histogram was constructed from 2500 traces without data selection (G_0 = 77481 nS).

3-3. 1 G_0 conductance regime

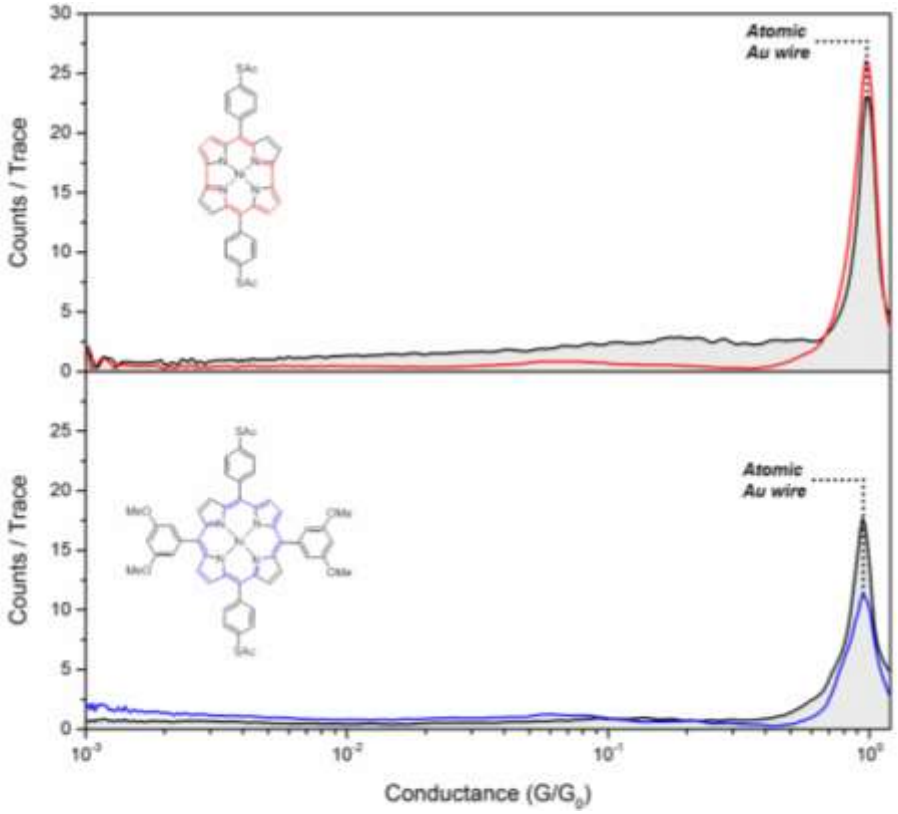

Supplementary Figure 5 | Logarithmically binned histograms of Ni(nor) and Ni(porph), obtained with 1μA/V preamplifiers at 100 mV using in air. The overlaid black histograms were obtained from freshly annealed Au(111) substrates prior to molecular deposition. No molecular signals in the high conductance regime. The sharp peak at 1 G_0 = 77481 nS arises from the formation of the atomic Au wire.

3-4. Ni(nor) 2D histogram data selection

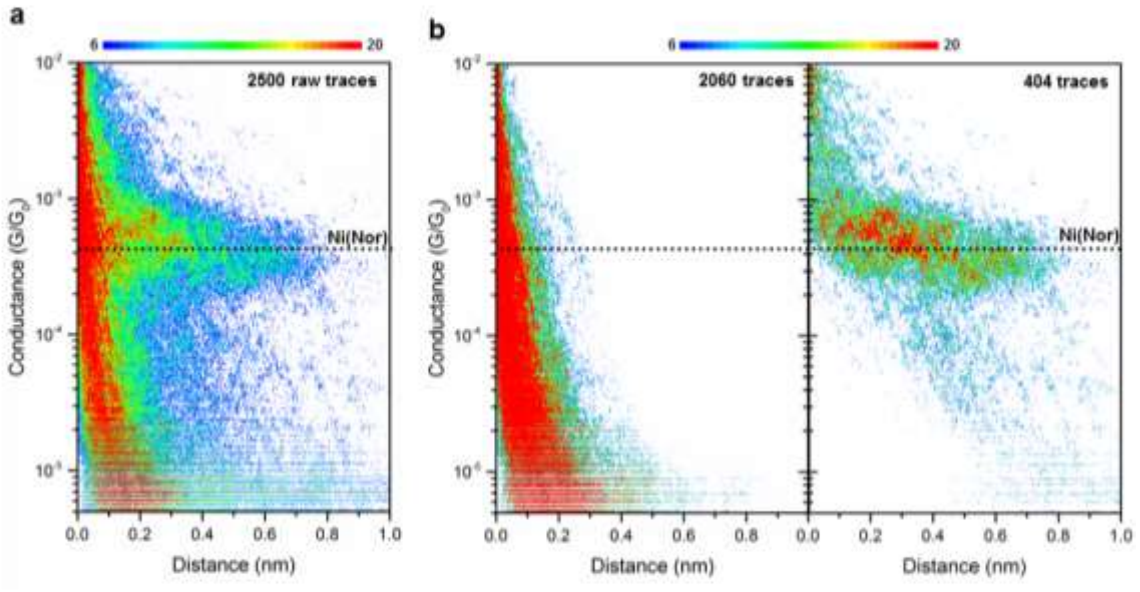

Supplementary Figure 6 | Data section of the conductance-distance traces. (**a**) 2D conductance-distance histogram of Ni(nor) at 100 mV in air, built with 2500 traces without data selection. (**b**) 2D histograms of the same dataset after removal of the STM-BJ traces following a conventional tunnelling decay. An automated subroutine was used to separate the 2060 traces reaching conductance values of $5 \cdot 10^{-5}$ G_0 in less than 0.45 nm. The remaining 404 traces (17.6 %) were employed to build the Figure 1b in the main text. This method was used to improve the visualization of the Ni(nor) plateau without distorting its essence. A $2.5 \cdot 10^{-3}$ nm bin size was used for the distance axes, while the conductance axes feature 150 bins per decade.

3-5. Additional 2D histograms (raw data)

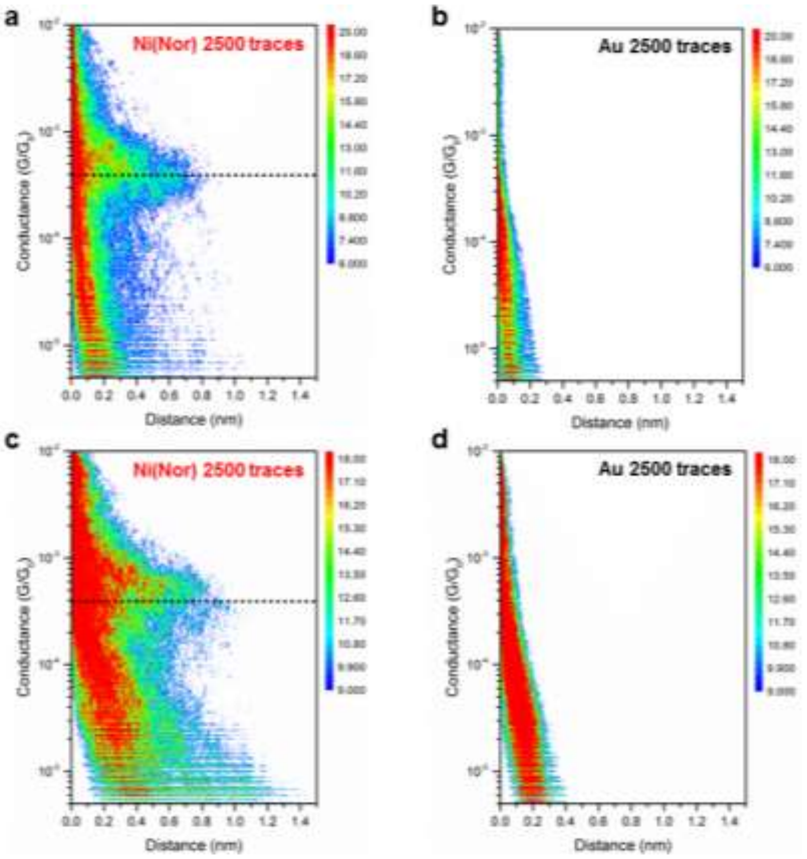

Supplementary Figure 7 | Conductance-distance 2D histograms, built from STM-BJ traces of (**a**) Ni(nor). (**b**) same Au(111) substrate prior to molecular deposition. (**c**) additional Ni(nor) sample. (**d**) same substrate before molecular deposition. Each individual histogram was constructed from 2500 traces without data selection. The conductance axes features 150 bins per decade (G_0 = 77481 nS), the distance axes features a bin size of 0.0025 nm.

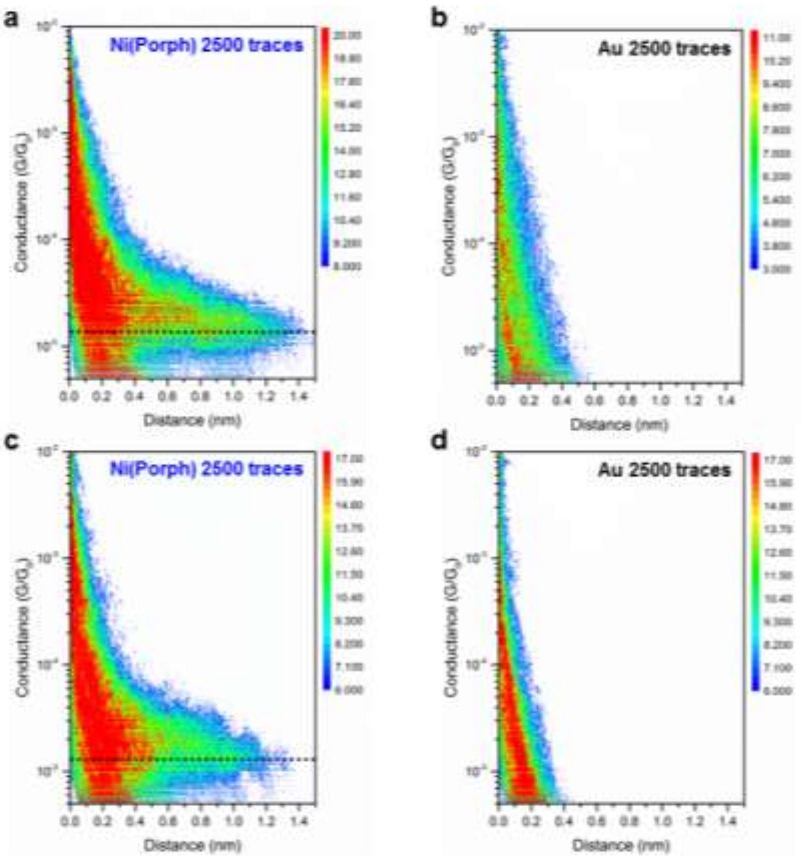

Supplementary Figure 8 | Conductance-distance 2D histograms, built from STM-BJ traces of (**a**) Ni(porph). (**b**) same Au(111) substrate prior to molecular deposition. (**c**) second Ni(porph) sample. (**d**) before molecular deposition. Each individual histogram was constructed from 2500 traces without data selection. The conductance axes features 150 bins per decade (G_0 = 77481 nS), the distance axes features a bin size of 0.0025 nm.

3-6. Working voltage influence on Ni(nor) and Ni(porph) junctions

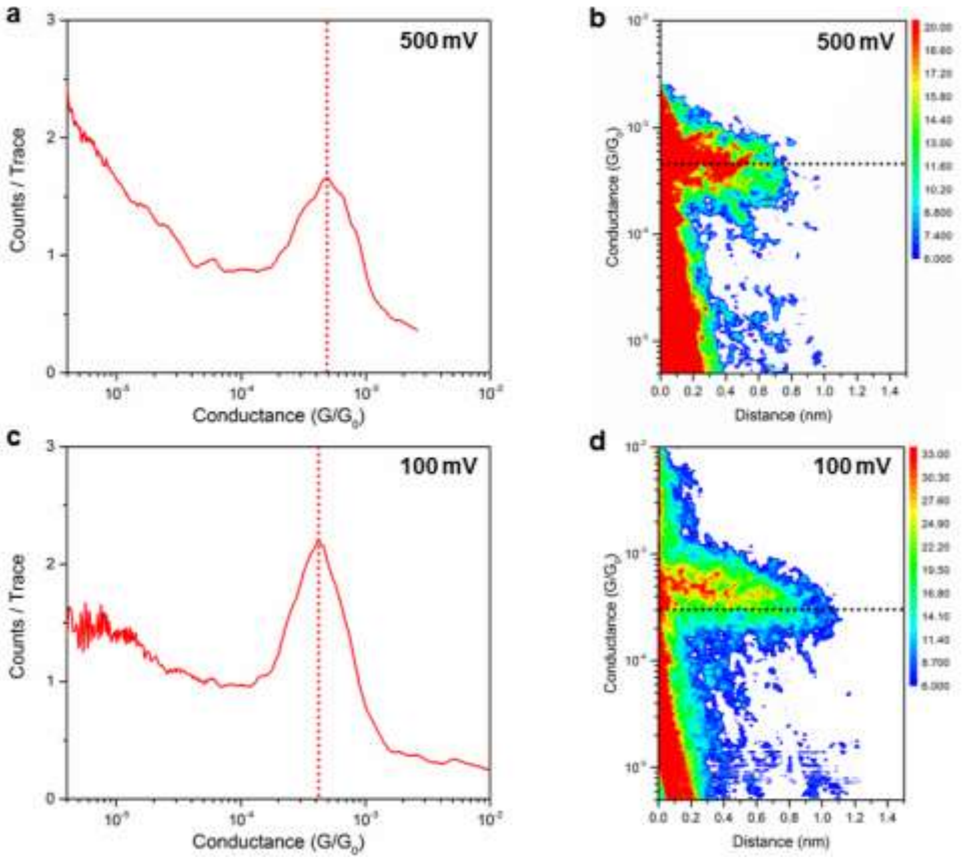

Supplementary Figure 9 | Working voltage influence on Ni(nor) and Ni(porph) junctions. (**a**) Conductance histogram of Ni(nor) STM-BJ traces at 500 mV in air. (**b**) Conductance-distance histogram built from the same dataset, (**c**) Same Ni(nor) sample measured at 100 mV. (**d**) 2D histogram of the same dataset. Histograms were constructed from 1000 traces without data selection. The conductance axes features 100 bins per decade (G_0 = 77481 nS), a 0.01 nm bin size was used for the distance axes.

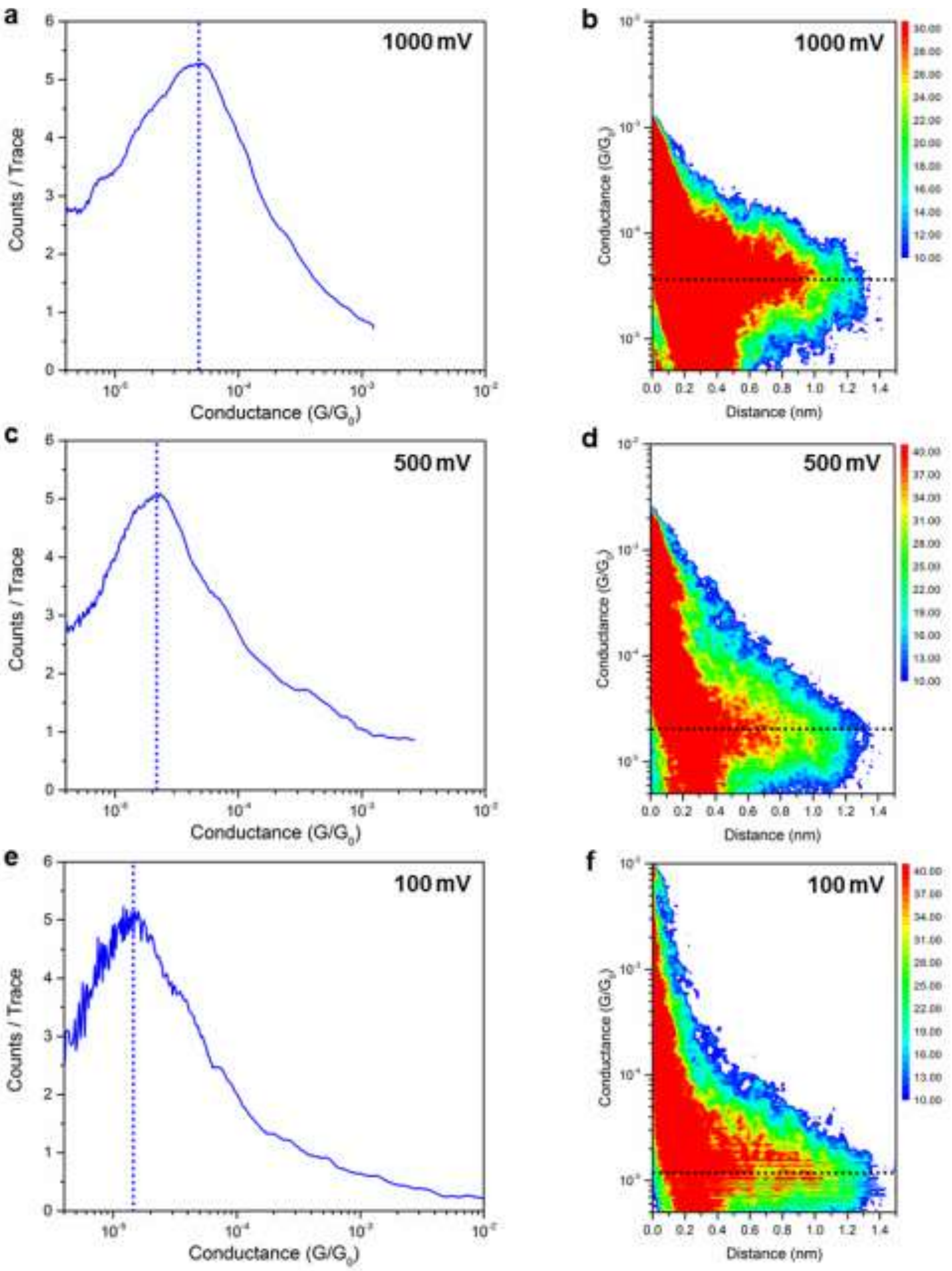

Supplementary Figure 10 | Working voltage influence on Ni(nor) and Ni(porph) junctions. (**a**) Conductance histogram of Ni(porph) STM-BJ traces at 1000 mV in air. (**b**) Conductance-distance histogram built from the same dataset. (**c**) Same sample measured at 500 mV. (**d**) 2D histogram of the same dataset. (**e**) Same Ni(porph) sample measured at 100 mV. (**f**) corresponding 2D histogram. Histograms were constructed from 1000 traces without data selection. The conductance axes features 100 bins per decade (G_0 = 77481 nS), a 0.01 nm bin size was used for the distance axes.

3-7. Structural details of Ni norcorrole and porphyrin complexes

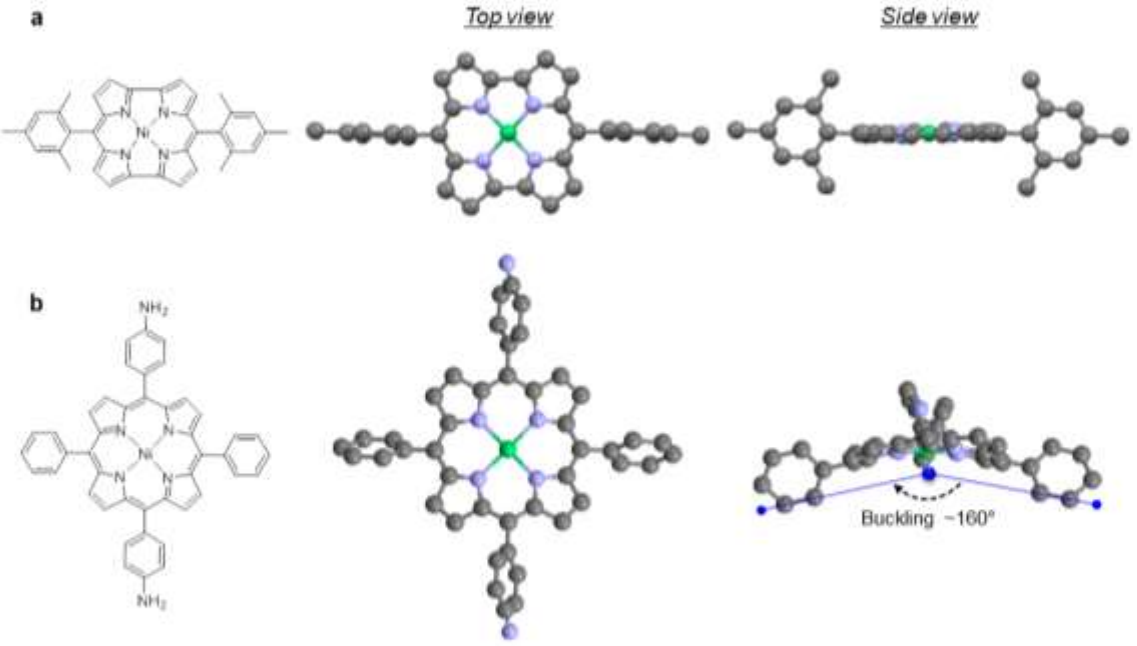

Supplementary Figure 11 | Representative examples of molecular structures previously reported in crystallographic studies, showing: (**a**) the highly planar configuration of the structurally constrained Ni norcorrole (CCDC 883624). [9] (**b**) buckled and twisted Ni porphyrin (CCDC 1180001).[10] Hydrogen atoms were removed for clarity. The rigid linear structure of Ni norcorrole may account for the comparably short molecular junctions *ca.*, 0.9 nm. The conformational richness of the porphyrin derivatives may increase its plasticity ultimately facilitating the formation of stable molecular junctions at longer tip-substrate distances.

Supplementary Note 4. Transmission studies

As mentioned in Supplementary Note 3, in the single-molecule junctions, the thioacetate groups (-SAc) of the Ni(nor) and Ni(porph) molecules are preserved and bind to Au electrodes through the S lone pair. Several tip motifs were investigated in the calculations.

The total conductance can be decomposed in terms of eigenchannel contributions. [11] For both Ni(nor) and Ni(porph), we find that only one transmission channel is dominant at the Fermi level and thus current is carried by a single channel. Interestingly, for both compounds the calculated spectra show a series of three sharp peaks close to each other, located in the 0.6~0.9 eV range for Ni(porph) and −0.4~−0.2 eV for Ni(nor). These sharp peak-dip structures reflect the crossing of different transmission channels, which are derived from quasi-degenerate molecular orbitals [12] of the aromatic and antiaromatic cores.

The energy of molecular projected self-consistent Hamiltonian (MPSH) [13] orbitals matches that of peaks or peak-dip features in the transmission spectra. The molecular gap is significantly smaller for the antiaromatic species. Notably, this arises from the conjugated cores and is not related to the metal atom. In both complexes the central Ni atom was replaced by two H atoms, whose positions were optimized while keeping all other coordinates constant. The transmission spectra of the complexes do not change significantly upon removal of the metal atom, but they exhibit fewer peaks or peak-dip features, consistent with the absence of Ni-derived states. The position of MPSH orbitals, displayed as solid diamonds in the Supplementary Figures shows a smaller gap in the antiaromatic species for both Ni- and Ni-free complexes.

We apply corrections to DFT-based conductance by taking into account errors in the DFT alignment of relevant frontier orbitals current.[14-17] For each species, we shift the position of the LUMO-derived resonance by an amount which includes self-energy corrections to the position of the isolated molecular level as well as screening effects at the junction. The magnitude of the self-energy corrections was obtained from total energy differences of the neutral and negatively charged species, making sure that the excitation populated the N-electron LUMO.[16,17] Screening effects at the metal interface were calculated using a classical image-charge model, taking into account the charge distribution of the LUMO and an image plane 1Å above the Au(111) surface.[15] We fitted the calculated transmission spectra to a lorentzian, which was then shifted in energy according to the corrections calculated above.

Corrected conductance values are in good agreement with experiment, as seen before for porphyrins.[14]

4-1. DFT transmission spectra with different tip structures

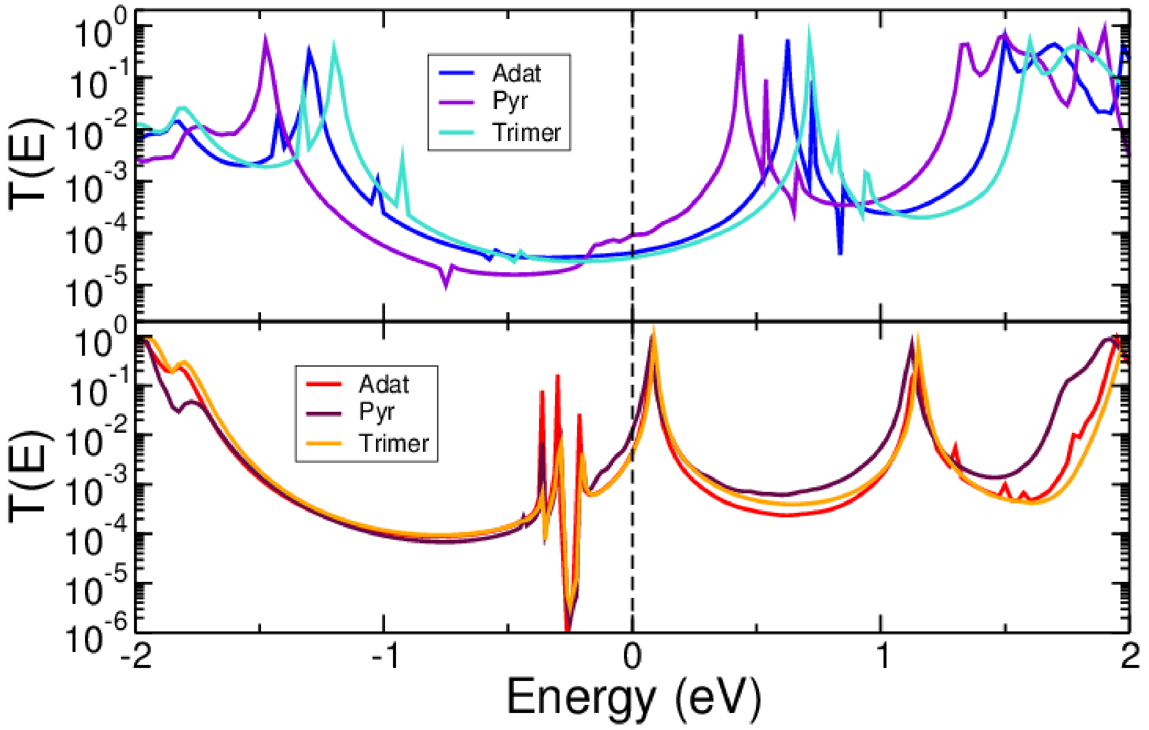

Supplementary Figure 12 | DFT transmission spectra with different tip structures. DFT transmission spectra for (top panel) Ni(porph) and (bottom panel) Ni(nor) complexes bound to gold *via* adatom, pyramidal or trimer tip motifs.

4-2. Eigenchannel decomposition

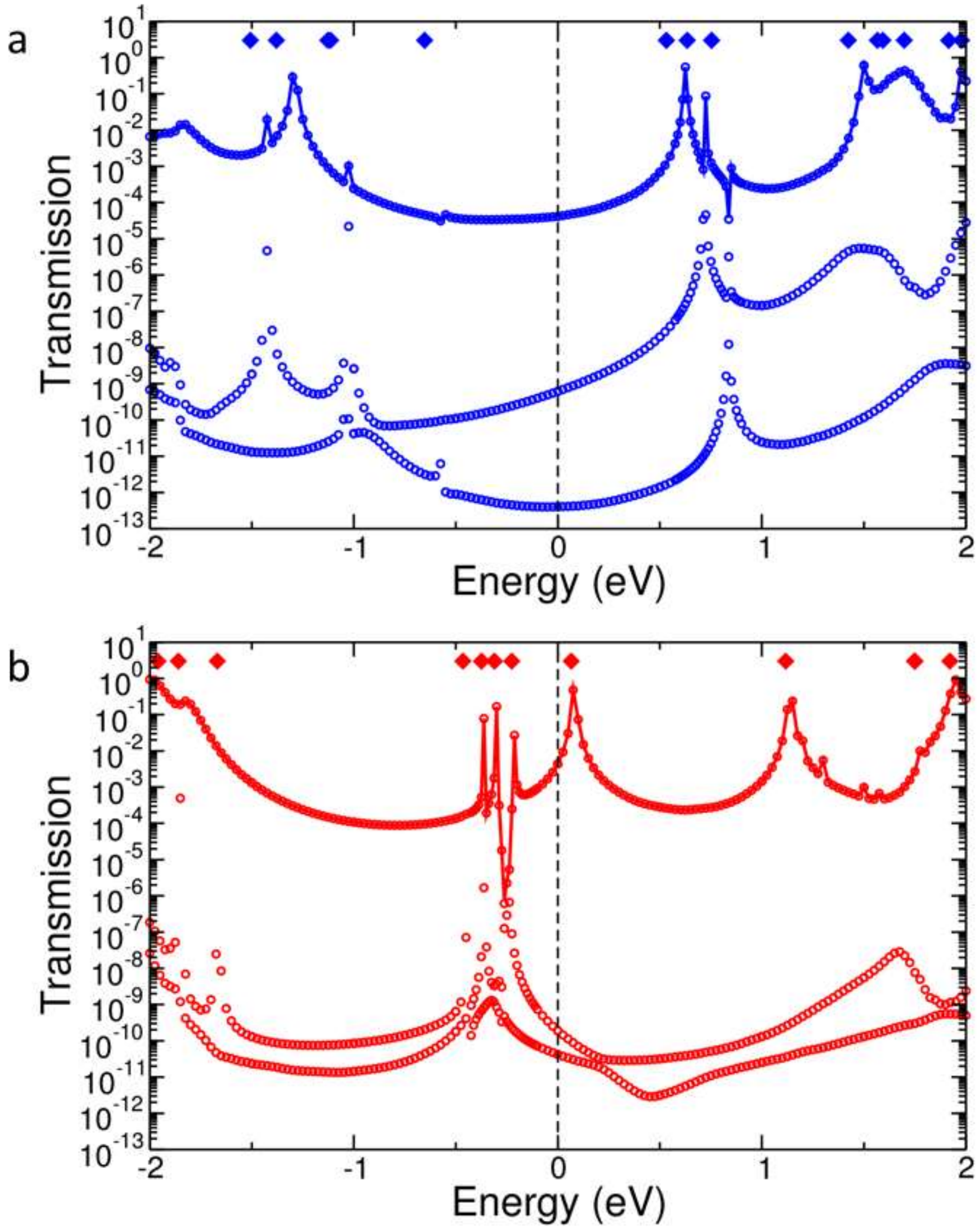

Supplementary Figure 13 | Eigenchannel decomposition. DFT transmission spectra of (**a**) Ni(porph) and (**b**) Ni(nor) with adatom tips. Solid diamonds indicate positions of MPSH orbitals. Total transmission (solid lines) and contribution of the 3 most conducting eigenchannels (open circles).

4-3. Molecular gap of aromatic and antiaromatic species

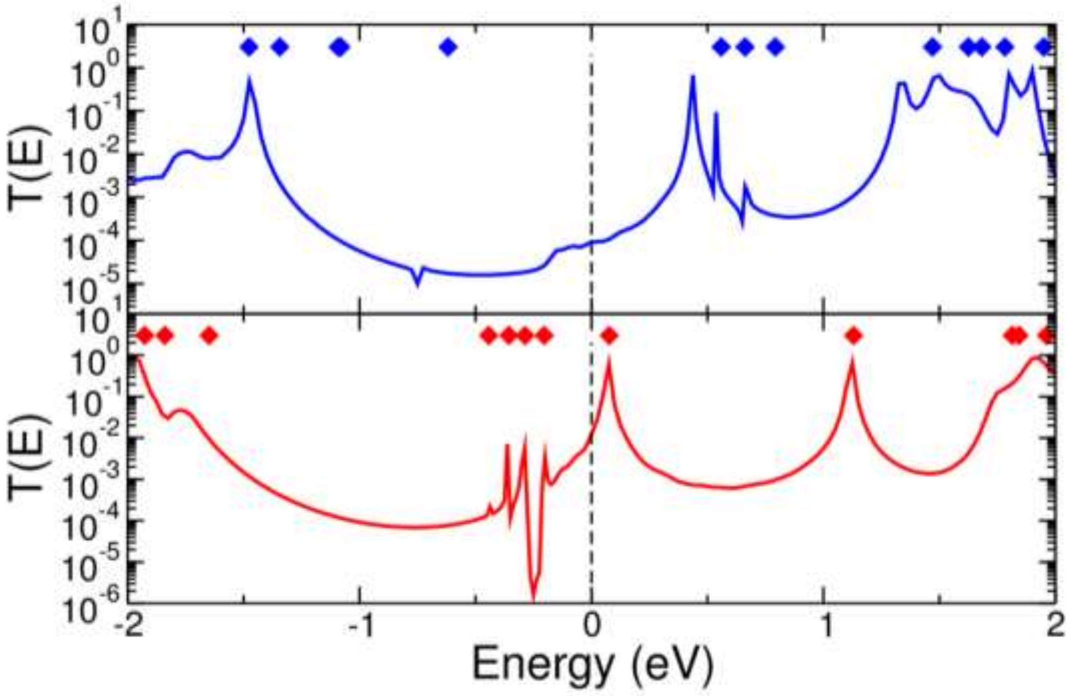

Supplementary Figure 14 | Molecular gap of aromatic and antiaromatic species. DFT transmission spectra for (top panel) Ni(porph) and (bottom panel) Ni(nor) complexes with pyramidal tip structures. Solid diamonds indicate positions of MPSH orbitals.

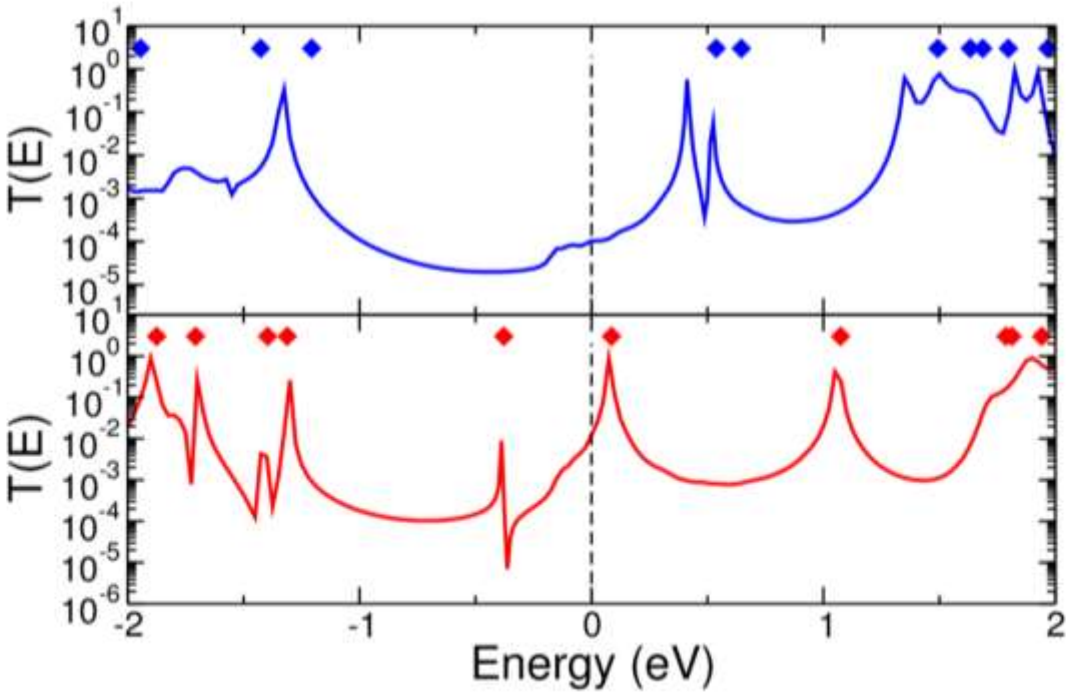

Supplementary Figure 15 | Molecular gap of metal-free aromatic and antiaromatic species. DFT transmission spectra for (top panel) H_2(porph) and (bottom panel) H_2(nor) metal-free complexes with pyramidal tip structures. Solid diamonds indicate positions of MPSH orbitals.

Supplementary Note 5. Corrections to the position of DFT molecular resonances

Transmission resonances were shifted in energy, while keeping the DFT-based peak height.

		Ni(nor)		Ni(porph)		Ratio
		E_{LUMO}-E_F	$G(E_F)$	E_{LUMO}-E_F	$G(E_F)$	G(nor)/G(porph)
**DFT**	**Pyramids**	0.08	1.2×10^{-2}	0.43	9.2×10^{-5}	130
	**Trimers**	0.09	4.2×10^{-3}	0.71	3.4×10^{-5}	124
	**Adatoms**	0.08	4.4×10^{-3}	0.63	4.2×10^{-5}	105
**Corrected**	**Pyramids**	0.68	1.4×10^{-4}	0.98	1.5×10^{-5}	9
	**Trimers**	0.37	2.6×10^{-4}	0.93	1.5×10^{-5}	17
	**Adatoms**	0.37	2.2×10^{-4}	0.87	1.6×10^{-5}	14
	**Average**	0.47	2.1×10^{-4}	0.93	1.5×10^{-5}	13
**Experiment**		0.48	4.2×10^{-4}	0.85	1.7×10^{-5}	25

Supplementary Table 1 | Comparison of calculated and measured conductance (in units of G_0) and molecular level offsets (in eV) of Ni(nor) and Ni(porph).

		Ni(nor)	Ni(porph)
		Γ	Γ
**Corrected**	**Pyramids**	20	9
	**Trimers**	13	11
	**Adatoms**	15	9
	**Average**	16	10
**Experiment**		18	13

Supplementary Table 2 | Comparison of calculated and experimentally determined electronic couplings (in units of meV).

Supplementary Note 6. Current-voltage experimental protocol

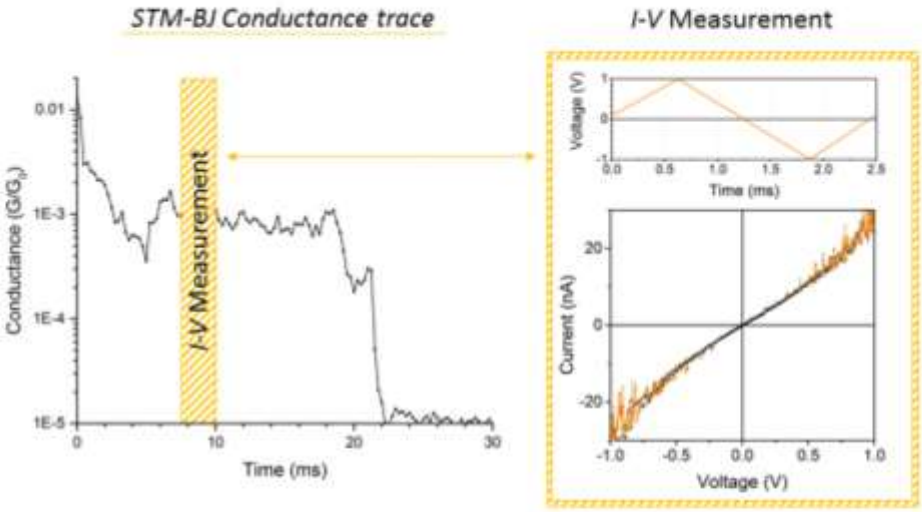

Supplementary Figure 16 | Current-voltage experimental protocol. Typical single-molecule *IV* measurement showing a molecular plateau in the conductance decay with a lifetime of approximately 20 ms. During the single-molecule regime the tip is held in position for 2.5 ms (delimited by an orange box) while the voltage is swept in the range ±1 V. A triangular waveform was employed to avoid sudden voltage changes that may lead to junction, or even molecular bond rupture. [2] After the *IV* response has been recorded, the break-junction process is recovered. The process is repeated and analysed statistically. Current-voltage profiles were included for the final analysis only when obtained from plateaus in the single-molecule regime for each complex *i.e.*, $[2\times10^{-4} – 10^{-3}]$ G_0 and $[4\times10^{-6} – 10^{-4}]$ G_0 for Ni(nor) and Ni(porph) respectively.

Supplementary Note 7. *I-V* analysis and single-level tunnelling model fitting

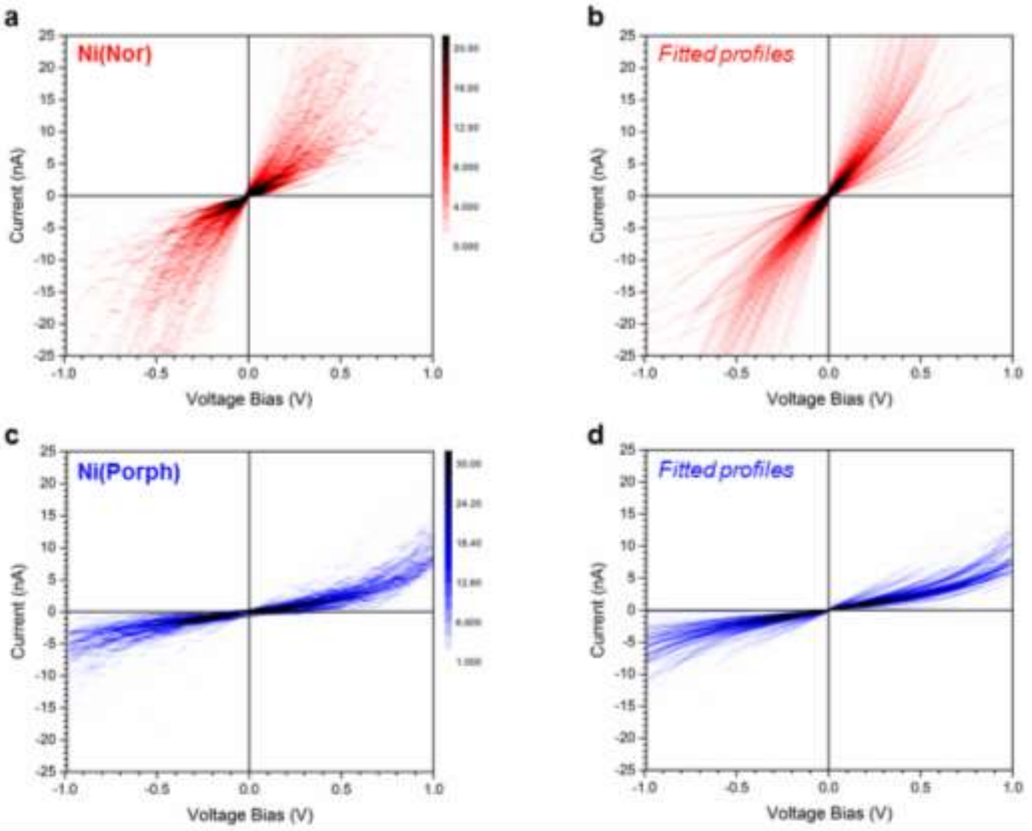

Supplementary Figure 17 | Analysis and fitting of *I-V*. (**a**) 2D histogram built from 216 experimentally obtained *I-V* profiles of Ni(nor). (b) 2D histogram containing the same number of profiles obtained from the fitting of the experimental data to a single-level tunnelling process. (c) 2D histogram built from 168 *IV* profiles of Ni(porph). (d) 2D histogram prepared with the theoretically fitted profiles assuming a single-level tunnelling process. This visual guide offers a straightforward method to confirm the goodness of the fitting conducted in this work. The fitted profiles accurately describe the IV profiles even at high voltage. Bin sizes of 0.02 V and 0.075 nA were employed.

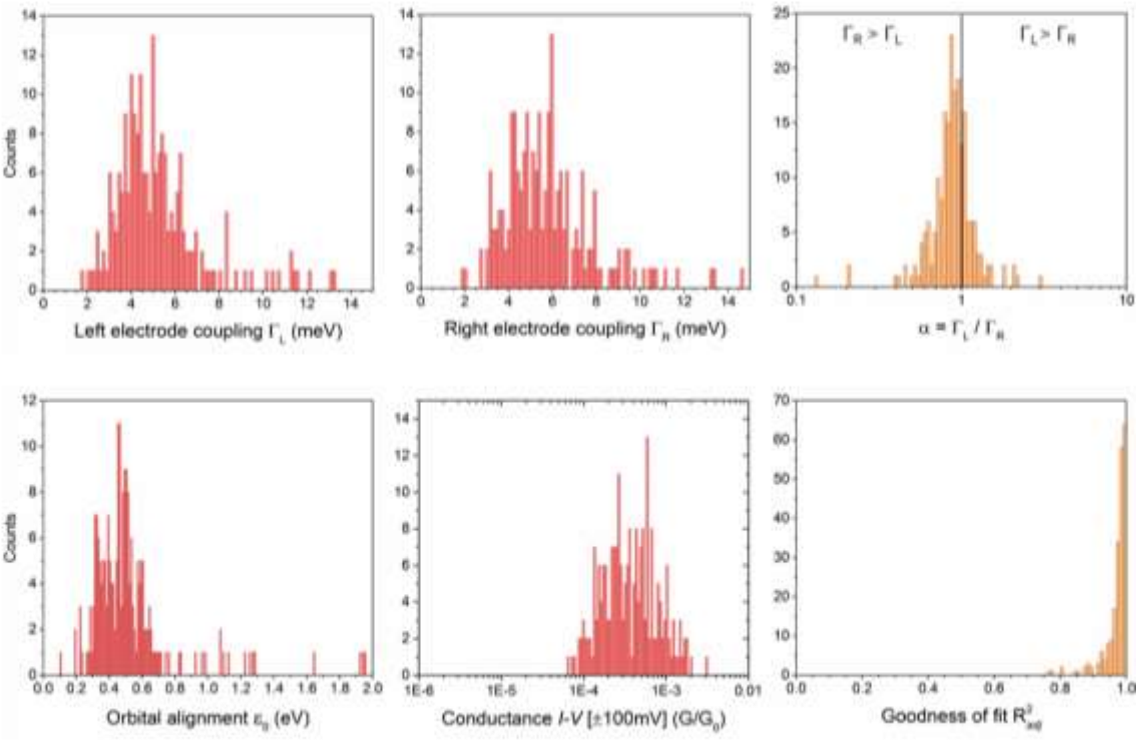

Supplementary Figure 18 | Analysis of *I-V* fitting results of Ni(nor). Statistics regarding the fitting of the *I-V* profiles of Ni(nor) to a single-level transport model. In clockwise order: degree of electronic coupling to left (Γ_L); and right (Γ_R) electrodes; coupling symmetry parameter ($\alpha = \Gamma_L/\Gamma_R$); energy difference between the molecular orbital and the Fermi level of the electrode; differential conductance obtained from the ± 100 mV (linear) regime of the *I-V* profile; goodness of fit as *adjusted* R^2. It should be noted that Γ (FWHM) in the main text corresponds to $2(\Gamma_L+\Gamma_R)$ in this Supplementary Figure18 and in Supplementary Figure 19.

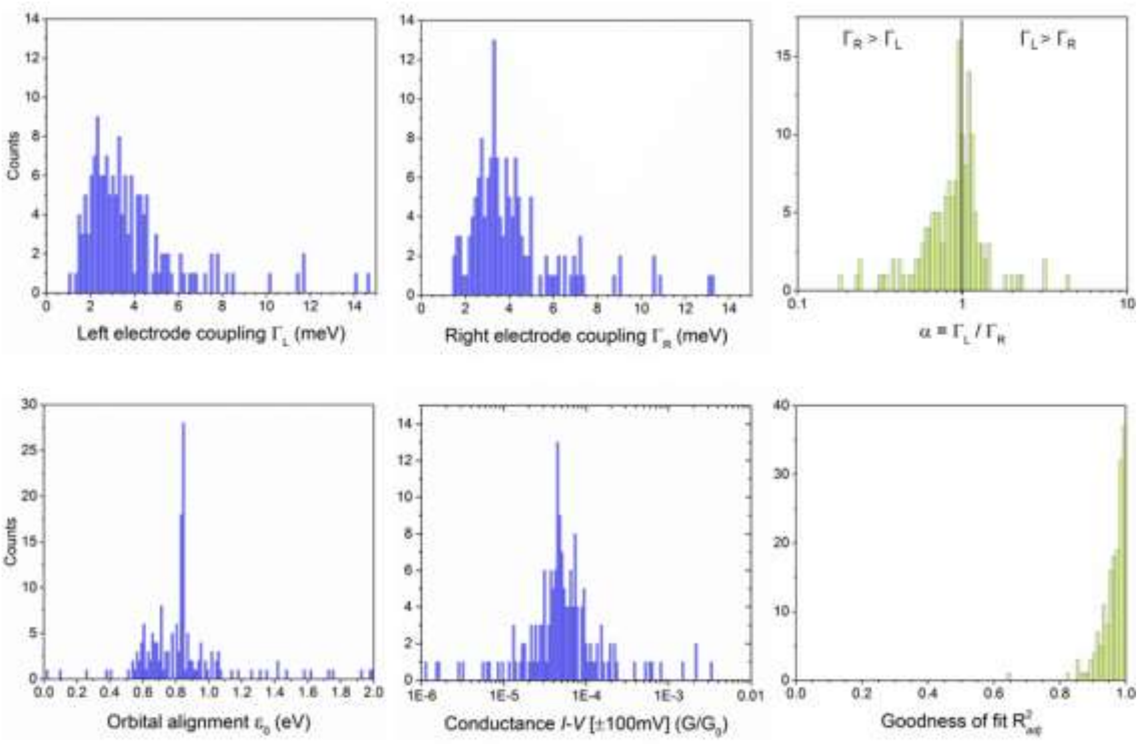

Supplementary Figure 19 | Analysis of *I-V* fitting results of Ni(porph). Statistics regarding the fitting of the *I-V* profiles of Ni(porph) to a resonant tunnelling model. In clockwise order: degree of electronic coupling to left (Γ_L); and right (Γ_R) electrodes; coupling symmetry parameter ($\alpha = \Gamma_L/\Gamma_R$); energy difference between the molecular orbital and the Fermi level of the electrode; conductance in the linear regime of the *I-V* profile (± 100mV); goodness of fit as *adjusted* R^2.

Supplementary Note 8. Ni(nor) STM-BJ studies under electrochemical control

8-1. Setup and experimental protocol

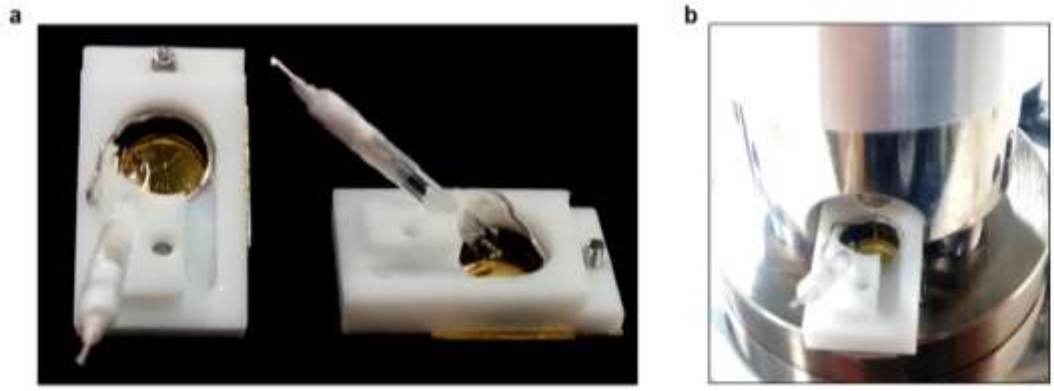

Supplementary Figure 20 | STM-BJ studies under electrochemical control. (**a**) Top and side view of the purpose-built 3D printable cell design used in the STM electrochemical modulation studies. The cell features a micro-sized Ag/AgCl reference electrode, and a coiled Pt wire (CE) in close proximity to the Au(111) substrate (WE_2). The 4-electrode setup is completed with a PDMS-coated electrochemically etched Au tip (WE_1). **(b**) Picture of the 4-electrode setup mounted on the STM stage, prior to the addition of the aqueous electrolyte.

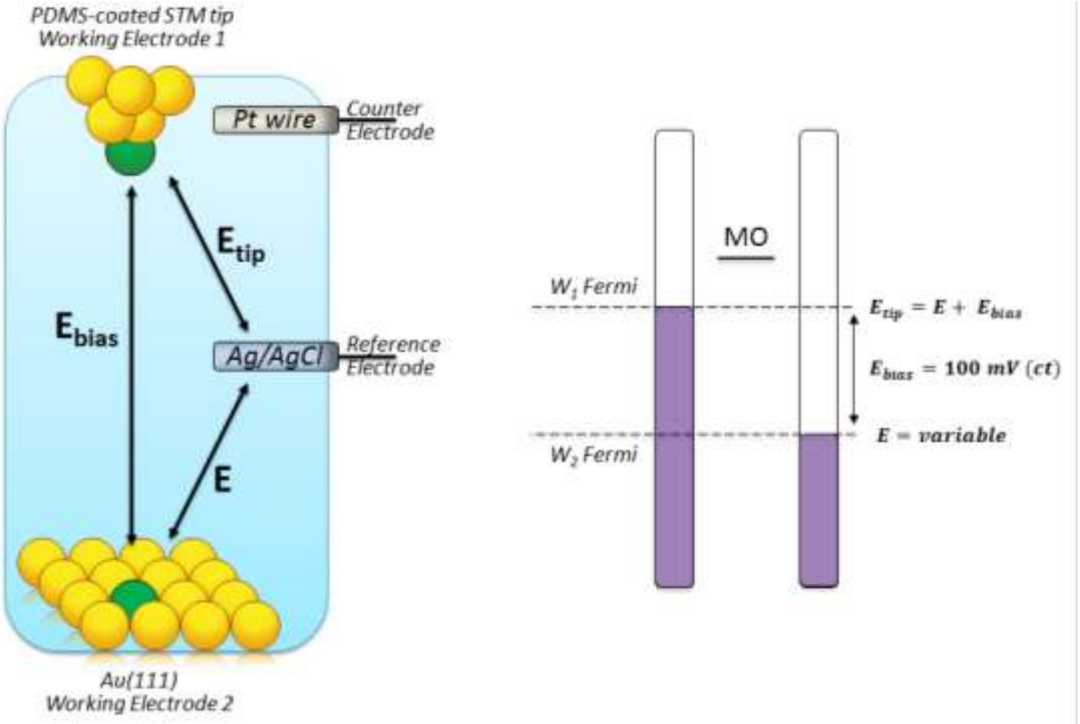

Supplementary Figure 21 | Schematic representation of the EC-STM setup. The 4-electrode arrangement consisted of: electrochemically-etched PDMS-coated gold tip (WE_1); Au(111) substrate (WE_2); a Pt counter electrode (CE); and a micro-sized Ag/AgCl (KCl_{sat}) reference electrode (RE). The tip-substrate voltage bias (E_{bias}) was kept constant at 100 mV. Break-junction experiments were repeated at different electrochemical ("gating") potentials (*E*). Changes in *E* modify the orbital alignment with respect with the Fermi level effectively altering the barrier shape. The substrates were then secured in place using a rubber O-ring and cell filled with ∼120 μL of pH 7. Experiments were paused and the electrolyte completely replaced every 15-20 minutes to avoid changes in the electrolyte concentration.

8-2. Electrochemical STM-BJ histograms of Ni(Nor)

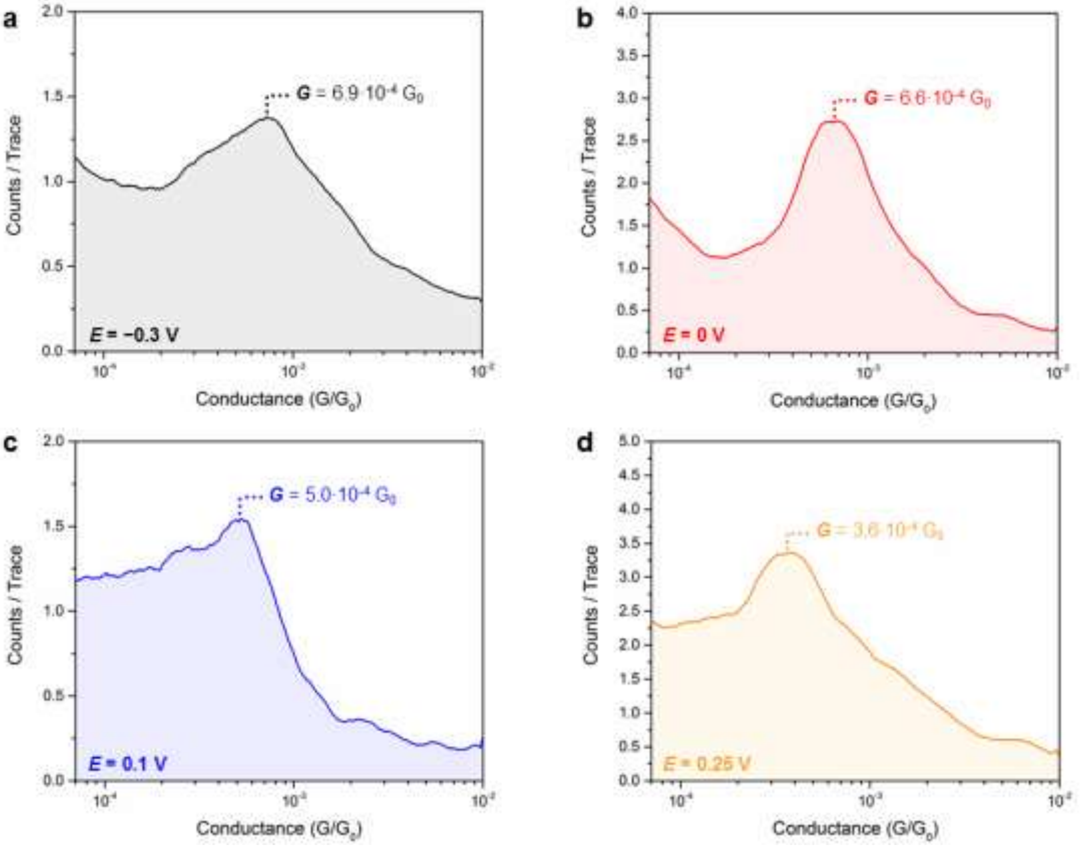

Supplementary Figure 22 | Electrochemical STM-BJ histograms of Ni(Nor). Logarithmically binned conductance histograms showing the most probable conductance value of Ni(nor) at different electrochemical potentials (*E*). Experiments were performed using a custom built STM electrochemical cell fitted with a Ag/AgCl reference electrode, in a pH 7 aqueous electrolyte (0.1 M, NaH_2PO_4/Na_2HPO_4). (G_0 = 77481 nS).

8-3. Additional EC-STM studies

Electrochemical modulation experiments were repeated to ensure the reproducibility of the observed modulation. Samples were prepared in an analogue way. In this case, a conventional STM liquid cell was employed, requiring the use of a Pt pseudo-reference electrode. Results were later referenced to an internal standard. Despite the increased uncertainty of these experiments, a comparable conductance modulation was observed upon reduction of the antiaromatic complex Ni(nor).

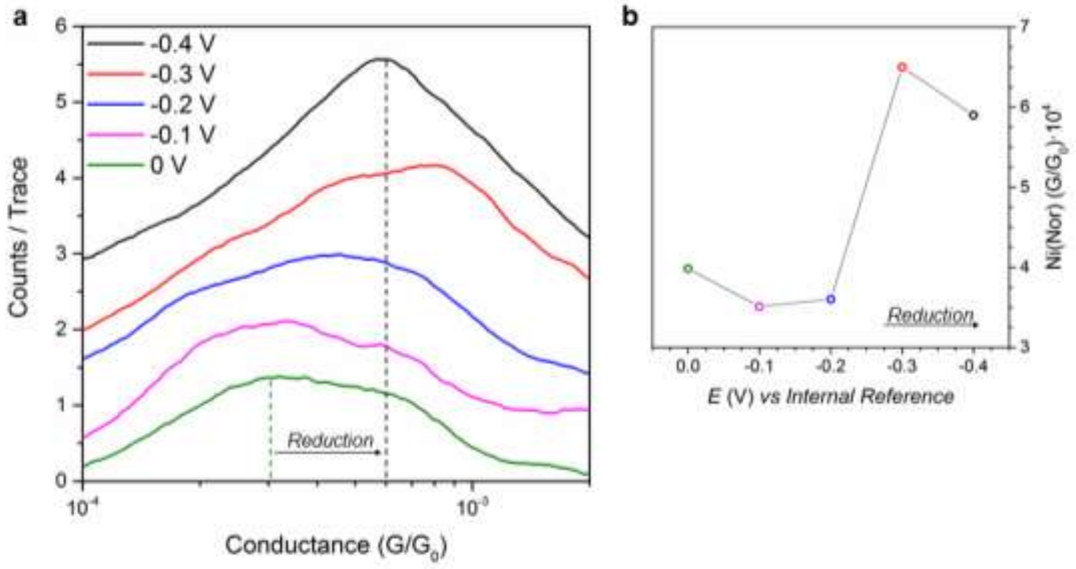

Supplementary Figure 23 | EC-STM studies using a Pt pseudo-reference electrode. Logarithmically binned histograms of Ni(nor) collected under different electrochemical voltage bias. Conductance modulation was observed under electrochemical control. Histograms were built from STM-BJ traces collected at a different electrochemical potential (*E*), while holding a constant tip-substrate bias of 0.1 V. Electrochemical potential was referenced against an internal reference.

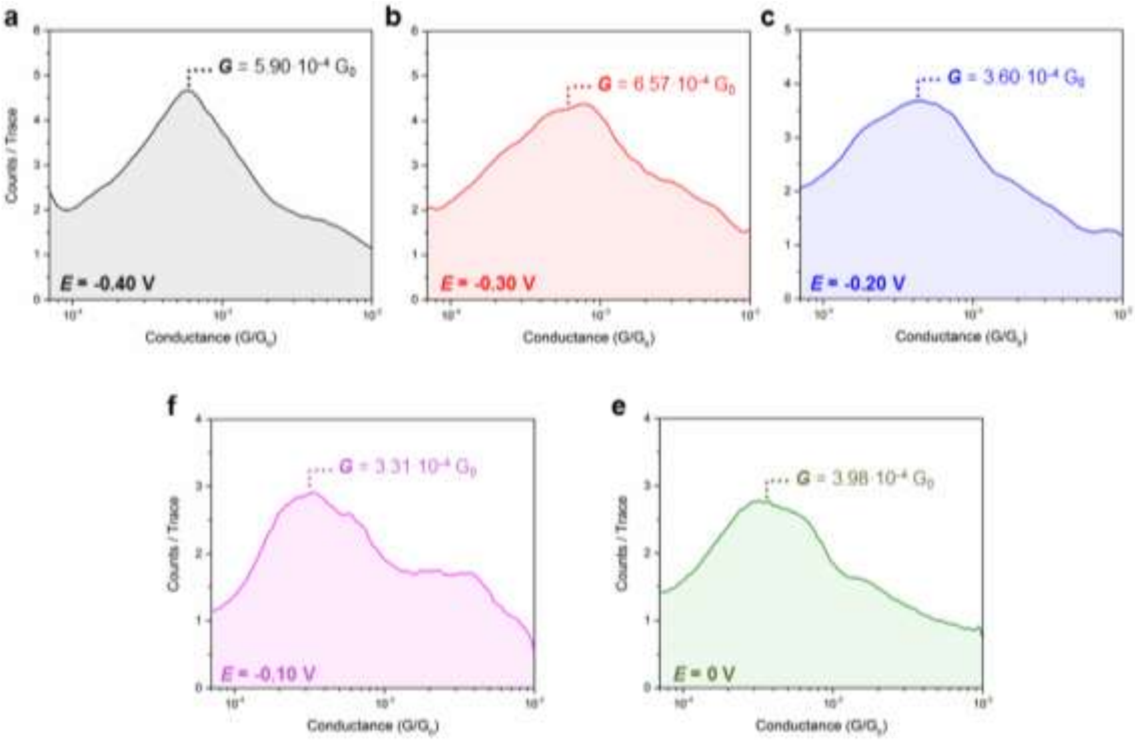

Supplementary Figure 24 | EC-STM studies using a Pt pseudo-reference electrode. Logarithmically binned conductance histograms showing the most probable conductance value of Ni(nor) at different electrochemical potentials (*E*). Experiments were performed using a commercially available STM liquid cell with Pt wire as a pseudoreference electrode in pH 7 aqueous electrolyte (0.1 M, NaH_2PO_4/Na_2HPO_4). Potential values are given against an internal reference. (G_0 = 77481 nS).

Supplementary Note 9. Ni(nor) STM-BJ studies under UHV conditions

Additional STM-BJ experiments of Ni(nor) were performed to check the reproducibility of the junction-formation and the robustness of the electronic transport properties. For the UHV-STM studies, samples were prepared in the manner as in the ambient STM-BJ studies, and transferred to an UHV-STM chamber. Details of the UHV-STM-BJ setup is described in the literature. [18] Conductance-distance traces measured in UHV (Supplementary Fig. 25a) corresponds well with those measured in air (Fig. 1 and Supplementary Fig. 7). The most probable single-molecule conductance was determined to be 4.6×10^{-4} G_0 (Supplementary Fig. 25b), which agrees well with that obtained in air (4.2×10^{-4} G_0). The molecule junctions were elongated up to >1 nm (Supplementary Fig. 25c). in UHV. This result rules out the possibility of the conductance difference being an artefact caused by measurements taking place perpendicularly to the norcorrole plane. Supplementary Figure 26a shows a 2D *I-V* histogram of Ni(nor), which was constructed from 1,000 *I-V* traces measured in UHV. The most probable single-molecule conductance at the bias voltage of 0.1 V was determined to be 5.2×10^{-4} G_0 (Supplementary Fig. 26b), which is in good agreement with the result of the conductance-distance measurement (Supplementary Fig. 25b).

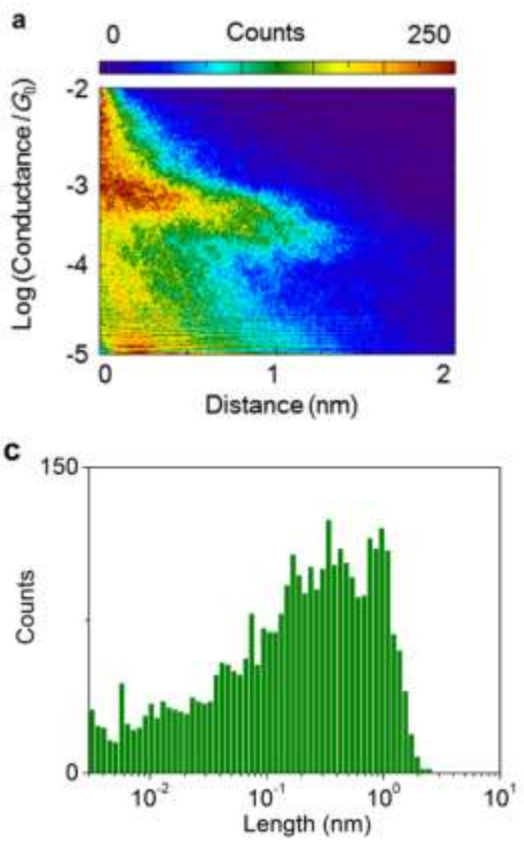

Supplementary Figure 25 | STM-BJ conductance studies of Ni(nor) under UHV conditions (**a**) Conductance-distance histogram of Ni(nor) measured at 100 mV under UHV. The histogram was constructed from 4,000 traces without data selection. The conductance axes features 100 bins per decade. A 0.01 nm bin size was used for the distance axes. (**b**) Conductance histogram built from the same dataset. The dotted and solid lines are raw and smoothed data, respectively. The arrow indicates a conductance peak position (4.6×10^{-4} G_0). (**c**) Length histogram built from the same dataset (10 bins per decade). The length of the molecule junction is defined as a plateau length within a conductance window form 10^{-2} to 10^{-4} G_0 in a conductance-distance trace.

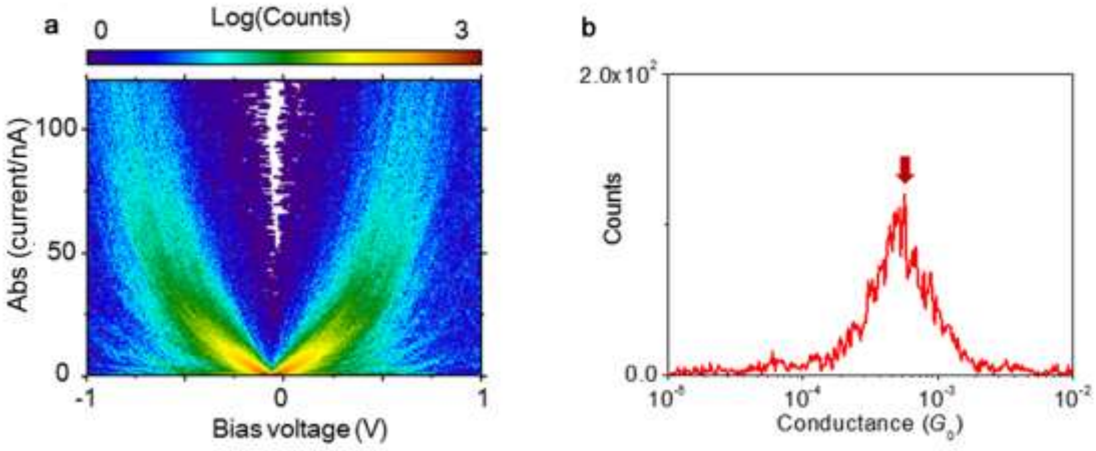

Supplementary Figure 26 | STM-BJ *I*-*V* studies of Ni(nor) under UHV conditions. (**a**) 2D *I*-*V* histogram of Ni(nor) constructed from 1,000 *I*-*V* traces measured under UHV. Bin sizes of 1.5 mV and 0.5 nA are used for the bias and absolute current/nA axis. (**b**) Conductance histogram constructed form 1,000 *I*-*V* traces measured under UHV. The conductance was calculated by current at the bias voltage of 0.1 V. A peak position (5.2×10^{-4} G_0) is indicated by an arrow in b.

Supplementary Note 10. Cyclic voltammogram of Ni(nor) on Au(111)

A Au(111) substrate was flame annealed by H_2 burner and then immersed in 1 mM decanethiol/ethanol solution for 2.5 hours to prepare decanethiol-self-assembled monolayer (SAM). After rinsing with a copious amount of ethanol, the Au(111) substrate was immersed in 20 mM Ni(nor)/dichloromethane solution for more than 4 days. Supplementary Figure 27 shows CVs of the SAM-modified Au(111) electrode in 0.1 M pH7 phosphate buffer solution with a scan rate of 100 mV s^{-1}. The cyclic voltammogram showed that the first electrochemical reduction, attributable to a reaction from Ni(nor) to Ni(nor) •−,19 appeared at −0.65 V vs Ag/AgCl.

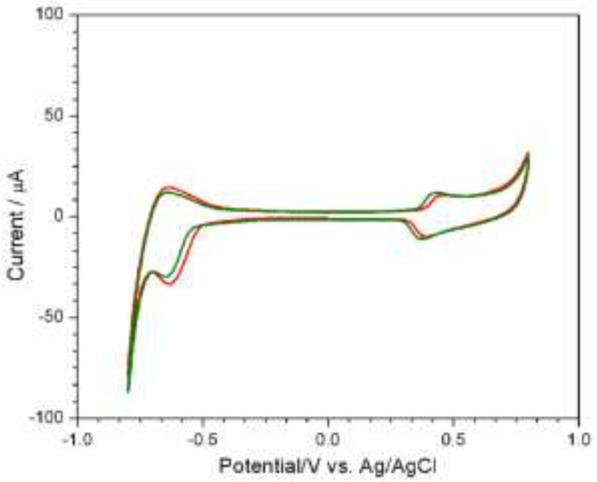

Supplementary Figure 27 | Cyclic voltammogram of Ni(nor) on Au(111). CVs of the SAM-modified Au(111) electrode in 0.1 M pH7 phosphate buffer solution.

Supplementary Note 11. Reduced HOMO-LUMO gap of antiaromatic molecules

The small HOMO-LUMO gap of the antiaromatic molecules can be intuitively explained by remarkable energetic destabilization in the monocyclic 4*n* π-electron system within Hückel's rule (see also main text) and understood by the theoretical calculation for a series of monocyclic π-conjugated molecules. Supplementary Figure 28 shows calculated HOMO-LUMO gaps for a series of planar monocyclic π-system of C_NH_N (N = 3~10) where N is number of the carbon atoms (π-electrons) in the monocyclic π-system. The monocyclic π-system exhibits gradual HOMO-LUMO gap closing with increasing the number of carbon atoms [19] with the obvious exception of N = 4 and 8 where the monocyclic π-system shares 4*n* π-electrons and is antiaromatic.

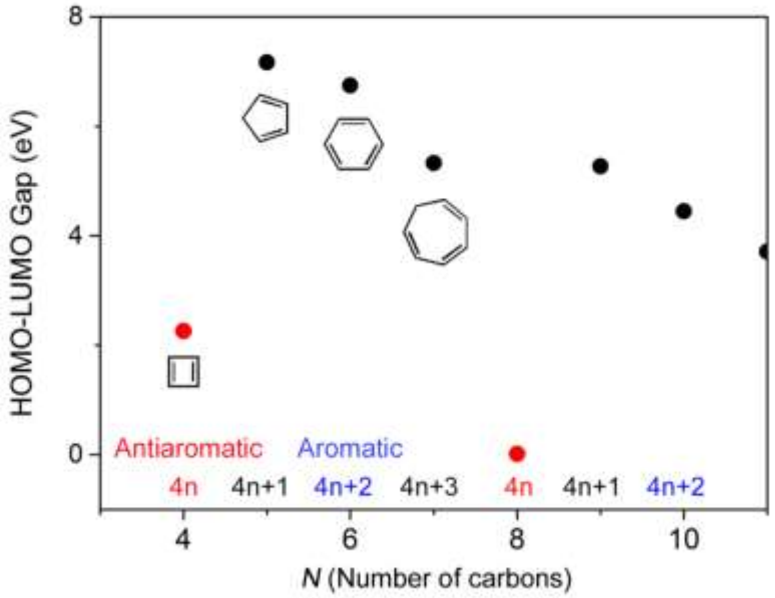

Supplementary Figure 28 | Calculated HOMO-LUMO gap for a series of monocyclic π-system of C_NH_N (N = 3~10) where N is number of the carbon atoms (*i.e.*, π-electrons) in the cyclic π-system. Insets are chemical structures for N = 4 (antiaromatic 4*n*), 5 (non-aromatic 4*n*+1), 6 (aromatic 4*n*+2), and 7 (non-aromatic 4*n*+3). The calculation was performed with the Gaussian 09 program [20] using the B3LYP functional and the 6-31G(d) basis set.

Supplementary References

1. Chen W., Li H., Widawsky J.R., Appayee C., Venkataraman L. & Breslow R. Aromaticity decreases single-molecule junction conductance. *J Am Chem Soc* **136**, 918-920 (2014).

2. Xie Z., Ji X.-L., Song Y., Wei M.-Z. & Wang C.-K. More aromatic molecular junction has lower conductance. *Chem Phys Lett* **639**, 131-134 (2015).

3. Mahendran A., Gopinath P. & Breslow R. Single molecule conductance of aromatic, nonaromatic, and partially antiaromatic systems. *Tetrahedron Lett* **56**, 4833-4835 (2015).

4. Quinn J.R., Foss F.W., Venkataraman L., Hybertsen M.S. & Breslow R. Single-Molecule Junction Conductance through Diaminoacenes. *J Am Chem Soc* **129**, 6714-6715 (2007).

5. Schneebeli S., *et al.* The Electrical Properties of Biphenylenes. *Org Lett* **12**, 4114-4117 (2010).

6. Park Y.S., *et al.* Contact Chemistry and Single-Molecule Conductance: A Comparison of Phosphines, Methyl Sulfides, and Amines. *J Am Chem Soc* **129**, 15768-15769 (2007).

7. González M.T., *et al.* Break-Junction Experiments on Acetyl-Protected Conjugated Dithiols under Different Environmental Conditions. *J Phy Chem C* **115**, 17973-17978 (2011).

8. Tour J.M., *et al.* Self-Assembled Monolayers and Multilayers of Conjugated Thiols, .alpha.,.omega.-Dithiols, and Thioacetyl-Containing Adsorbates. Understanding Attachments between Potential Molecular Wires and Gold Surfaces. *J Am Chem Soc* **117**, 9529-9534 (1995).

9. Ito T., Hayashi Y., Shimizu S., Shin J.Y., Kobayashi N. & Shinokubo H. Gram-scale synthesis of nickel(II) norcorrole: the smallest antiaromatic porphyrinoid. *Angew Chem Int Ed Engl* **51**, 8542-8545 (2012).

10. Hayvalı M., Gündüz H., Gündüz N., Kılıç Z. & Hökelek T. Synthesis and characterization of unsymmetrically tetrasubstituted porphyrin and their nickel (II) complexes with the crystal structure of 5,15-bis(4-aminophenyl)-10, 20-diphenylporphyrinatonickel(II). *J Mol Struct* **525**, 215-226 (2000).

11. Paulsson M. & Brandbyge M. Transmission eigenchannels from nonequilibrium Green's functions. *Phys Rev B* **76**, 115117 (2007).

12. Bergfield J.P., Barr J.D. & Stafford C.A. The Number of Transmission Channels Through a Single-Molecule Junction. *ACS Nano* **5**, 2707-2714 (2011).

13. Brandbyge M., Mozos J.-L., Ordejón P., Taylor J. & Stokbro K. Density-functional method for nonequilibrium electron transport. *Phys Rev B* **65**, 165401 (2002).

14. Liu Z.F., *et al.* Control of single-molecule junction conductance of porphyrins via a transition-metal center. *Nano Lett* **14**, 5365-5370 (2014).

15. Smith N.V., Chen C.T. & Weinert M. Distance of the image plane from metal surfaces. *Phys Rev B* **40**, 7565-7573 (1989).

16. Cheng Z.L., *et al.* In situ formation of highly conducting covalent Au-C contacts for single-molecule junctions. *Nat Nanotechnol* **6**, 353-357 (2011).

17. Vazquez H., *et al.* Probing the conductance superposition law in single-molecule circuits with parallel paths. *Nat Nanotechnol* **7**, 663-667 (2012).

18. Fujii S., *et al.* Rectifying Electron-Transport Properties through Stacks of Aromatic Molecules Inserted into a Self-Assembled Cage. *J Am Chem Soc* **137**, 5939-5947 (2015).

19. Malloci G., Cappellini G., Mulas G. & Mattoni A. Electronic and optical properties of families of polycyclic aromatic hydrocarbons: A systematic (time-dependent) density functional theory study. *Chem Phys* **384**, 19-27 (2011).

20. Frisch M.J., *et al.* Gaussian 09, Revision B.01. (2009).
